# Congenital urethral diverticulum in an adult male patient presenting with anejaculation

**DOI:** 10.1016/j.eucr.2023.102463

**Published:** 2023-06-25

**Authors:** Kholoud Alabassi, Abdulla Al-Naimi, Osama Abdalfattah, Tarek Ibrahim

**Affiliations:** Department of Surgery, Urology Section, Hamad Medical Corporation, Doha, 3050, Qatar

**Keywords:** Urethral diverticulum, Anejaculation, Case report

## Abstract

Male urethral diverticulum is an uncommon condition typically caused by previous surgeries, inflammation, or trauma. There are very few case reports of primary male urethral diverticulum, with only one report linking it to ejaculatory problems. In this report, we present a rare case of congenital male urethral diverticulum who presented with lower urinary tract symptoms and anejaculation that was successfully treated through open urethral diverticulectomy.

## 1 Introduction

Congenital anterior urethral diverticula are rare in male patients. This condition was first described and reported in 1906 by Watts.^1^ Since then, some cases have been reported. Here, we report a case of a congenital anterior urethral diverticulum in an adult male who presented with voiding difficulty, congenital penoscrotal swelling, and anejaculation.

## 2 Report of the case

A 27-year-old male presented to the urology clinic complaining of scrotal swelling since birth, anejaculation, intermittent dysuria, straining, frequency, and urgency. He had no history of urological surgery. Physical examination revealed a fluctuating, approximately 10-mm central cystic lesion at the penoscrotal junction; no other abnormalities were observed.

**Fig. 1:**
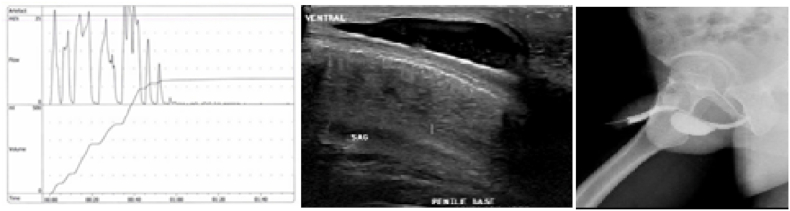
Preoperative investigations; Uroflowmetry, ultrasound and Urethrogram.

The urine flow rate was measured, and the pattern flow results are presented in Fig. 1.

Ultrasound examination of the urethra revealed a well-defined, oval-shaped cystic lesion, which was compressible during scanning and contained fluid with thick, moving debris, seen anteriorly at the base of the penis (Fig. 1).

A urethrogram showed ventral outpouching at the penile urethra measuring 4 × 2.5 cm. The bulbar urethra was intact (Fig. 1).

**Fig. 2:**
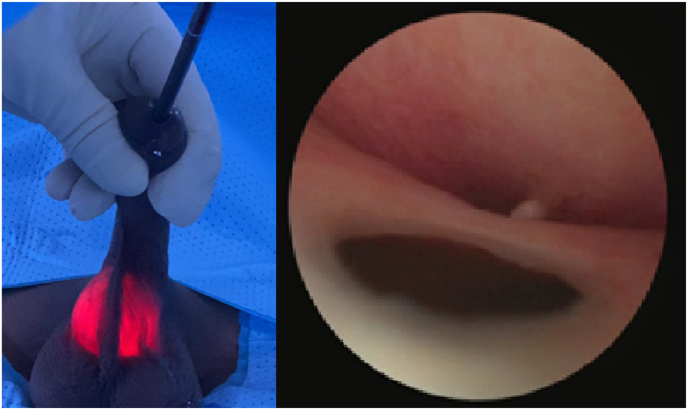
Cystoscopy.

Diagnostic cystoscopy revealed an opening in the ventral wall of the urethra, leading to a large diverticulum at the proximal penile urethra (Fig. 2). The opening was approximately 3 mm in diameter (admitting a 9F flexible ureteroscope).

**Fig. 3:**
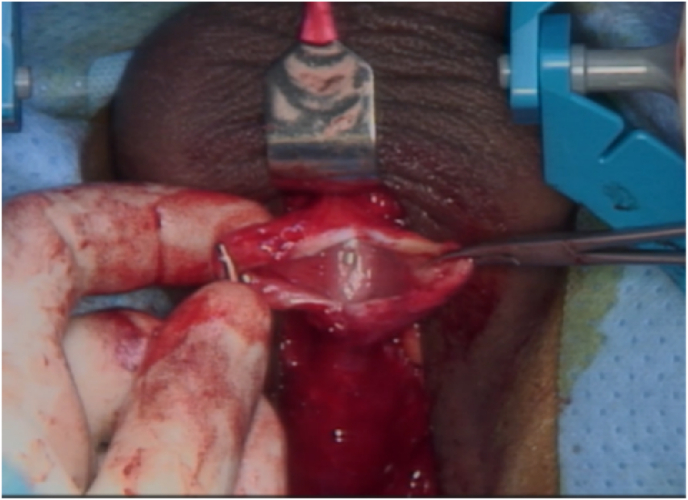
The diverticulum is opened showing the urethral defect on the ventral aspect of the urethra.

Diverticulum excision was performed through a perineal skin incision. The opening of the diverticulum was identified ventrally at the proximal penile urethra, and the diverticular sac was excised (Fig. 3). The diverticulum opening was trimmed and closed without tension over a 16F urethral catheter using 5-0 Vicryl, followed by reinforcement with the dartos layer.

**Fig. 4:**
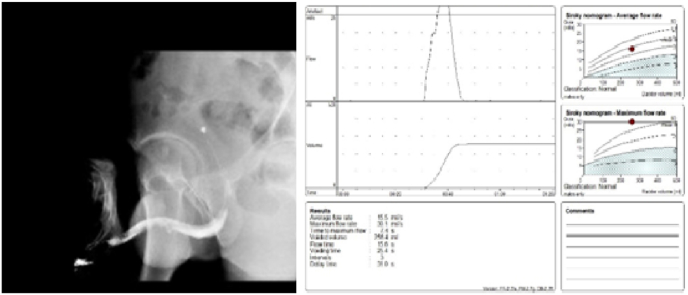
Post-operative urethrogram and uroflowmetry.

A peri-catheter urethrogram was obtained at 3 weeks postoperatively, which revealed no contrast extravasation or fistula formation (Fig. 4). The postoperative flow rate is depicted in Fig. 4.

During the subsequent follow-up, at 3 months and 6 months, the patient reported no difficulty in voiding or ejaculation.

## 3 Discussion

Although many theories about the formation of congenital anterior urethral diverticula and various proposed etiological mechanisms have been described in the literature, the embryological origin remains uncertain.^1^

While some have considered the anterior urethral valve and diverticula as a single entity, others have differentiated the congenital anterior urethral valve by the coverage of the corpus spongiosum while the anterior urethral diverticulum protruded through the spongiosum and was covered only by skin, as seen in our case.^2^

The congenital anterior diverticula shape can be either saccular with wide communication or spherical with a narrow neck.^1^ The diverticulum in our patient had a saccular shape with a narrow neck.

Anterior urethral diverticula can be found on the ventral aspect of the urethra between the bulbous and mid-penile urethra. In this case, the diverticulum was located ventrally in the proximal penile urethra.

Patients with urethral diverticula may be asymptomatic or symptomatic. The symptoms include lower urinary tract symptoms, dysuria, and hematuria. Although manifestations such as infertility are extremely rare among patients with congenital urethral diverticula, this has been reported in the literature.^3^

Magnetic resonance imaging has been used to evaluate male urethral diverticula. However, fluoroscopic modalities, in conjunction with urethral ultrasonography, are the best diagnostic techniques for confirming and characterizing urethral diverticula,^4^ providing excellent details.

Urethral diverticula can be managed endoscopically or through open surgery. The endoscopic approach is associated with a high risk of recurrence; therefore, an open approach is usually preferred.^5^

Dartos flaps can reinforce the repair to reduce the risk of urethrocutaneous fistula.^5^ In our case, a urethral opening was found in the penile urethra, where the spongiosum was deficient anteriorly; therefore, we reinforced the repair with a Dartos flap.

Postoperative complications were reported in the literature, including infection, urinary retention, recurrence, urethrocutaneous fistula and urethral stricture formation within two years.^5^ Our patient did not experience any of these complications within the first 6 months.

## 4 Conclusion

Congenital male urethral diverticulum is a rare condition that should be contemplated as a possible diagnosis when patients exhibit scrotal swelling and symptoms related to the lower urinary tract. Anejaculation may also be a potential complication, leading to infertility. Surgical excision of the diverticulum cures the symptoms and regains ejaculation.

## Declaration of competing interest

There are no conflicts of interest.
